# Inhaled protein/peptide-based therapies for respiratory disease

**DOI:** 10.1186/s40348-016-0044-8

**Published:** 2016-04-20

**Authors:** Robert C. Fellner, Shawn T. Terryah, Robert Tarran

**Affiliations:** Cystic Fibrosis and Pulmonary Diseases Research and Treatment Center, University of North Carolina, 7102 Marsico Hall, 125 Mason Farm Road, Chapel Hill, NC 27599-7248 USA; Spyryx Biosciences, 801-9 Capitola Drive, Durham, NC 27713 USA; Department of Cell Biology and Physiology, University of North Carolina, Chapel Hill, NC 27599 USA

**Keywords:** CFTR, Biotherapies, Neutrophil elastase (NE), Inflammation, Goblet cell metaplasia, Nebulization, Aerosolization, Omalizumab, Alpha-1-antitrypsin (AAT), Pulmozyme, Mucociliary clearance, PLUNC, ENaC, BPIFA1

## Abstract

Asthma, chronic obstructive pulmonary disease (COPD), and cystic fibrosis (CF) are all chronic pulmonary diseases, albeit with different etiologies, that are characterized by airflow limitation, chronic inflammation, and abnormal mucus production/rheology. Small synthetic molecule-based therapies are commonly prescribed for all three diseases. However, there has been increased interest in “biologicals” to treat these diseases. Biologicals typically constitute protein- or peptide-based therapies and are often more potent than small molecule-based drugs. In this review, we shall describe the pros and cons of several different biological-based therapies for respiratory disease, including dornase alfa, a recombinant DNAase that reduces mucus viscosity and short palate lung and nasal epithelial clone 1 (SPLUNC1)-derived peptides that treat Na^+^ hyperabsorption and rebalance CF airway surface liquid homeostasis.

## Introduction

For hundreds of years, the pulmonary system has been used to deliver pharmacologically active compounds to the body [47]. The lungs allow for efficient drug delivery as they have a large surface area and are well vascularized [35]. For example, inhaled nicotine is readily absorbed across the pulmonary epithelia into the bloodstream where it can exert its psychotropic effects on the brain [5]. Conversely, for many peptides/proteins, an inability to cross the respiratory epithelium after inhaled delivery may actually be advantageous as it would result in a high ratio of lung to systemic bioavailability and thus would reduce off-target effects [25]. As a case in point, inhaled antibiotics achieve far higher concentrations with far fewer side effects than orally delivered antibiotics [55, 62]. The majority of drugs in use today are classed as “small molecules.” That is, organic chemicals typically bind to their receptor to elicit a response [41, 57]. Since these molecules are often extremely durable, until metabolized by the liver and/or cleared by the kidney, they can have side effects in other organs [22]. In contrast, biological therapeutics, including proteins (e.g., antibodies, enzymes) and peptides, show considerable promise and are emerging as alternatives to small molecule-based drugs [19]. Some protein-based therapies have failed in the clinic, since they are more labile than small molecules and are prone to proteolytic degradation in the blood [32, 39]. However, protein-based therapies show great promise for many types of respiratory disease since they can be delivered to the target organ directly by inhalation. Additionally, whilst small molecules typically have nanomolar potency, biologicals often have picomolar to femtomolar potency due to their increased ability to bind to their protein target with high affinity. This increased binding is achieved due to the ability of proteins and peptides to change their conformation during binding to better fit the binding pocket in their receptor [2]. This review concentrates on asthma, cystic fibrosis (CF), and chronic obstructive pulmonary disease (COPD), three respiratory diseases typified by airflow limitations and poor alveolar gas exchange.

## Review

### Characteristics of asthma, CF, and COPD

Asthma is typified by chronic airway inflammation caused by a combination of environmental and genetic factors [44]. Symptoms include airway hyperreactivity, airway narrowing, goblet cell metaplasia/mucus hyperproduction, and eosinophilia [13, 16]. Asthma is typically treated by a combination of β-agonists and corticosteroids to relax smooth muscle and reduce inflammation, with a subset of patients being non-responsive to these medications, suggesting an unmet need for new asthma therapies [31].

CF is a multi-organ inherited disease, caused by mutations in the CF gene product, the cystic fibrosis transmembrane conductance regulator (CFTR), a cAMP-regulated anion channel [53]. The lack of functional CFTR and subsequent epithelial sodium channel (ENaC) hyperactivation result in Cl^−^ hyposecretion and Na^+^ hyperabsorption, respectively, that combine to dehydrate airway surfaces [3, 12]. CF lung disease is characterized by the accumulation of dehydrated/viscous mucus, leading to chronic infection/inflammation goblet cell metaplasia, neutrophilia, and bronchiectasis [26, 38]. The positive effects from nebulization of hypertonic saline or mannitol by CF patients indicate that rehydration therapy is a viable therapeutic mechanism for the treatment of CF lung disease [14, 46].

COPD is the third leading cause of death world-wide and can have a number of different causes, with tobacco exposure being the most common [10]. COPD is typified by alveolar destruction, coughing/chronic mucus production, chronic inflammation, and protease imbalance which lead to irreversible airflow limitation and a progressive loss of lung function [30]. COPD treatments include inhaled bronchodilators and steroids [23]. In severe cases, long-term oxygen therapy is required but to date, there are no effective therapies to reverse the disease, even after smoking cessation.

### Antibody therapies

Monoclonal antibodies (mAbs) are now a well-established class of FDA-approved drugs used to treat asthma (e.g., omalizumab/Xolair™) [25]. Therapeutic mAbs are typically full-length IgGs that have a molecular weight of ∼150 kDa [42, 52]. Unlike previous generations of mAbs, most mAbs currently used in clinical trials are fully human in origin and are produced using either transgenic animals or phage display technology, which helps to reduce immunogenicity, increase effector function, and prolong their serum half-life [9, 52]. Whilst we only highlight what we feel are the advantages and disadvantages regarding this type of therapeutic, we direct the readers to several other excellent reviews that cover this area in more detail [4, 11, 43, 58].

mAbs offer several advantages over small molecules. First, they bind with high affinity and specificity to a wide variety of proteins. Second, they are relatively stable, allowing them to remain active for long periods of time. Third, since their breakdown products are amino acids, they are not converted into toxic metabolites [8, 52]. Whilst inhalation offers an attractive route for delivery of mAbs, perhaps surprisingly, mAbs are delivered parenterally for respiratory disorders, with the inhalation route yet to make it into the clinic. However, mAbs retain their physical and immunological properties after aerosolization, suggesting that it is only a matter of time before mAb inhalation is utilized therapeutically [25, 37, 42].

When considering the route of administration for mAbs, matching the delivery route to the therapeutic target’s location is paramount. This was highlighted by studies using the mAb omalizumab to treat allergic asthma. Omalizumab is a chimeric mAb that specifically binds to and neutralizes IgE, thereby preventing its interaction with mast cells and the subsequent release of histamine and other inflammatory mediators [1]. Unlike intravenous administration, pulmonary delivery of omalizumab failed to attenuate the response to inhaled allergens in asthmatic subjects [15, 17]. The observed lack of efficacy in the aerosolization trial was likely a failure of the pulmonary route to deliver sufficient systemic omalizumab to neutralize free systemic IgE [25]. Another possible disadvantage of mAbs compared to small molecules is their molecular weight. Most small molecules are hundreds of dalton to a few kilodaltons whereas mAbs are in excess of 100 kDa, which makes inhaled delivery less efficient, but would be less of a problem for systemic delivery [51].

A final consideration regarding the use of mAbs as respired therapeutics that was also illustrated by the aerosolized omalizumab trial is immunogenicity. Although inhaled omalizumab was generally well-tolerated, one test subject developed serum IgG and IgA antibodies against omalizumab. This finding led the authors to speculate that inhaling full-length mAbs may be more immunogenic than administering them parentally [15]. However, the degree of aggregation of aerosolized omalizumab after nebulization was not evaluated, and since aggregated proteins are known to be highly immunogenic, this may have been the cause [52]. Regardless, we agree that the development of drug-specific IgG and IgA antibodies are a concern that need to be monitored and that further studies are needed to better understand the immunogenicity of inhaled mAbs.

### Peptides and proteins

**Fig. 1:**
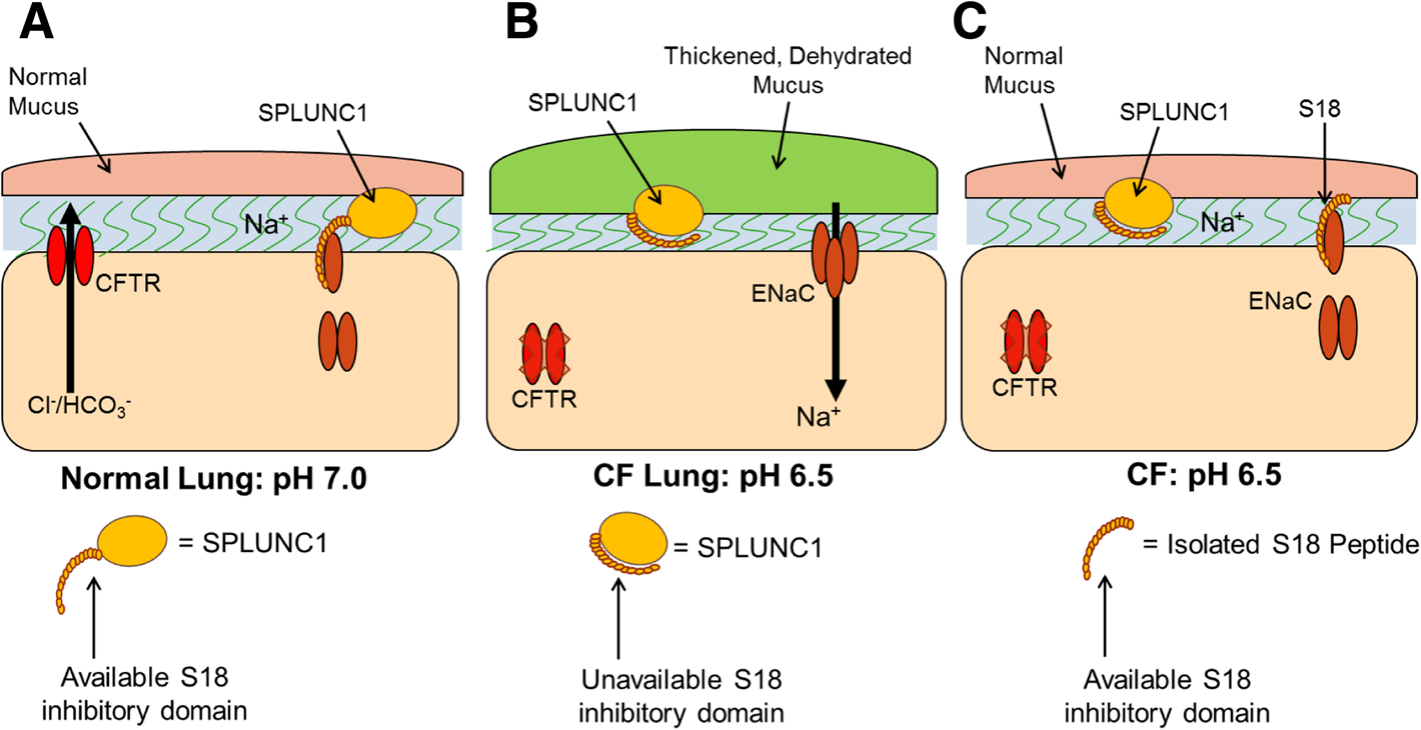
Rationale for SPLUNC1-derived peptide therapy for CF lung disease. **a** In normal airways, bicarbonate secretion through CFTR maintains ASL pH at ~7.0. At this pH, secreted SPLUNC1 can bind to ENaC, leading to internalization and inhibition of the channel. This helps maintain airways hydration and mucus clearance. **b** In CF airways, the acidic ASL, caused by a lack of bicarbonate secretion through dysfunctional CFTR, causes SPLUNC1 to adopt an inappropriate conformation, where the ENaC inhibitory domain (also known as the S18 region) cannot bind to ENaC, leading to Na^+^ hyperabsorption and ASL dehydration. **c** S18-derived peptides are pH-independent and can inhibit ENaC to reduce Na^+^ absorption and help normalize airway hydration/mucus clearance in acidic CF airways

Short palate lung and nasal epithelium clone 1 (SPLUNC1) is a ~25-kDa protein that contains an ENaC inhibitory domain, which for historical reasons was called the S18 region [29]. Unlike traditional ion channel antagonists which block ENaC’s pore, SPLUNC1 inhibits ENaC by inducing endocytosis [54] (Fig. 1a). Since SPLUNC1 fails to regulate ENaC in the CF lung (Fig. 1b) [21], Spyryx Biosciences is currently developing a SPLUNC1-derived peptide, which functions in CF airways as an ENaC inhibitor (Fig. 1c) [18, 59]. This restoration of CF airway surface liquid (ASL) hydration is predicted to (i) improve mucociliary clearance and (ii) decrease infection/inflammation [7, 34]. Additionally, these peptides are intrinsically disordered so they are heat stable. Another advantage of intrinsically disordered proteins/peptides is that they achieve a greater contact area with their target protein, thus maximizing binding efficiency [6]. S18-derived peptides are protease resistant, do not freely cross the respiratory epithelium, and do not reach the kidney to induce the hyperkalemia, as seen with small molecule ENaC antagonists like amiloride [27, 28]. Chronic inhalation therapy using these peptides could produce local immunogenicity and irritation, but given that SPLUNC1-derived peptides are naturally occurring in normal but not CF lungs, immunogenicity would seem unlikely [29]. A limitation of this type of therapeutic is that it would only ameliorate CF lung disease and would not treat other CF-affected organs.

Both CF and COPD airways exhibit increased neutrophil elastase (NE) activity, which has the potential to damage the lung and also to cleave and activate ENaC, exacerbating mucus dehydration and further reducing mucociliary clearance [24, 40, 45, 50]. Alpha-1-antitrypsin (AAT) is an endogenous NE inhibitor which is predicted to improve pulmonary function by blocking NE. Kamada Inc. has an inhaled biological based on human AAT, which is in phase 2 clinical trials for treatment of CF [20, 33]. A potential limitation of AAT is that in addition to NE, several other proteases including cathepsins and metalloproteases are also upregulated in CF/COPD which may also contribute to the lung damage but would not be blocked by AAT.

CF and COPD airways are characterized by high levels of DNA [49] and actin [60] in the lung lumen, which are released by necrotic neutrophils [36]. Excess DNA and actin adversely alter mucus rheology and increase viscosity, leading to decreased mucociliary clearance [48]. Therefore, another approach to increase mucociliary clearance in CF and COPD lungs is to decrease mucus viscosity by cleaving extracellular DNA. Dornase alfa is a recombinant version of human Dnase1 protein that is used as a therapeutic for CF [61]. Dnase1 cleaves extracellular DNA in the lung lumen leading to reduced DNA length/concentration and, therefore, reduced sputum viscosity. Pulmozyme is a recombinant version of human Dnase1 marketed by Genentech for the treatment of CF. Pulmozyme is administered via nebulization and has been shown to reduce the incidence of CF infections [56].

## Conclusions

Biotherapies constitute the fastest growing sector of approved drugs, but their delivery via the lung remains a nascent field. It is increasingly clear, however, that inhaled biological therapeutics can offer some strong advantages over traditional therapeutics including increased potency, reduced systemic availability, and potentially, a longer duration of action. There are several biological drugs that are either approved or in the development pipeline, and here, we have highlighted some that we feel are showing promise to succeed where traditional small molecules and the parenteral delivery route have failed. These examples make it clear that this is an exciting field that warrants future investigation.

## Abbreviations

AAT: alpha-1-antitrypsin
ASL: airway surface liquid
CF: cystic fibrosis
CFTR: cystic fibrosis transmembrane conductance regulator
COPD: chronic obstructive pulmonary disease
ENaC: epithelial sodium channel
mAb: monoclonal antibody
NE: neutrophil elastase
SPLUNC1: short palate lung and nasal epithelium clone 1

## References

[1] (2002) Omalizumab: anti-IgE monoclonal antibody E25, E25, humanised anti-IgE MAb, IGE 025, monoclonal antibody E25, Olizumab, Xolair, rhuMAb-E25. BioDrugs 16:380–386 10.2165/00063030-200216050-00009 12408744

[2] Alberts B, Johnson A, Lewis J, Raff M, Roberts K, Walter P (2002). Molecular biology of the cell.

[3] Althaus M (2013). ENaC inhibitors and airway re-hydration in cystic fibrosis: state of the art. Curr Mol Pharmacol.

[4] Antoniu SA (2013). Monoclonal antibodies for asthma and chronic obstructive pulmonary disease. Expert Opin Biol Ther.

[5] Benowitz NL, Hukkanen J, Jacob P 3rd (2009) Nicotine chemistry, metabolism, kinetics and biomarkers. Handb Exp Pharmacol 192:29–60 10.1007/978-3-540-69248-5_2 PMC2953858 19184645

[6] Berlow RB, Dyson HJ, Wright PE (2015). Functional advantages of dynamic protein disorder. FEBS Lett.

[7] Boucher RC (2004). New concepts of the pathogenesis of cystic fibrosis lung disease. Eur Respir J.

[8] Chames P, Van Regenmortel M, Weiss E, Baty D (2009). Therapeutic antibodies: successes, limitations and hopes for the future. Br J Pharmacol.

[9] Chan AC, Carter PJ (2010). Therapeutic antibodies for autoimmunity and inflammation. Nat Rev Immunol.

[10] Chapman KR, Mannino DM, Soriano JB, Vermeire PA, Buist AS, Thun MJ, Connell C, Jemal A, Lee TA, Miravitlles M, Aldington S, Beasley R (2006). Epidemiology and costs of chronic obstructive pulmonary disease. Eur Respir J.

[11] Charriot J, Vachier I, Halimi L, Gamez AS, Boissin C, Salama M, Cucu-Jarjour A, Ahmed E, Bourdin A (2016). Future treatment for asthma. Eur Respir Rev.

[12] Collawn JF, Matalon S (2014). CFTR and lung homeostasis. Am J Physiol Lung Cell Mol Physiol.

[13] Damera G, Panettieri RA (2011). Does airway smooth muscle express an inflammatory phenotype in asthma?. Br J Pharmacol.

[14] Elkins MR, Robinson M, Rose BR, Harbour C, Moriarty CP, Marks GB, Belousova EG, Xuan W, Bye PT, National Hypertonic Saline in Cystic Fibrosis Study G (2006). A controlled trial of long-term inhaled hypertonic saline in patients with cystic fibrosis. N Engl J Med.

[15] Fahy JV, Cockcroft DW, Boulet LP, Wong HH, Deschesnes F, Davis EE, Ruppel J, Su JQ, Adelman DC (1999). Effect of aerosolized anti-IgE (E25) on airway responses to inhaled allergen in asthmatic subjects. Am J Respir Crit Care Med.

[16] Fahy JV, Dickey BF (2010). Airway mucus function and dysfunction. N Engl J Med.

[17] Fahy JV, Fleming HE, Wong HH, Liu JT, Su JQ, Reimann J, Fick RB, Boushey HA (1997). The effect of an anti-IgE monoclonal antibody on the early- and late-phase responses to allergen inhalation in asthmatic subjects. Am J Respir Crit Care Med.

[18] Fellner RC, Terryah S, Oddo J, Taylor J, Arendshorst W, Tarran R, and Christensen D (2015) SPLUNC1 peptide-derivatives with increased efficacy and decreased renal side effects in vitro and in vivo. In: North American Cystic Fibrosis Conference. Phoenix, Arizona

[19] Fosgerau K, Hoffmann T (2015). Peptide therapeutics: current status and future directions. Drug Discov Today.

[20] Gaggar A, Chen J, Chmiel JF, Dorkin HL, Flume PA, Griffin R, Nichols D, and Donaldson SH (2015) Inhaled alpha-proteinase inhibitor therapy in patients with cystic fibrosis. J Cyst Fibros. 15(2):227-33. 10.1016/j.jcf.2015.07.009 PMC4993024 26321218

[21] Garland AL, Walton WG, Coakley RD, Tan CD, Gilmore RC, Hobbs CA, Tripathy A, Clunes LA, Bencharit S, Stutts MJ, Betts L, Redinbo MR, Tarran R (2013). Molecular basis for pH-dependent mucosal dehydration in cystic fibrosis airways. Proc Natl Acad Sci U S A.

[22] Golan DE, Tashjian AH, Armstrong EJ, Armstrong AW (2007). Principles of pharmacology: the pathophysiologic basis of drug therapy.

[23] Gordon E, Lazarus SC (2009). Management of chronic obstructive pulmonary disease: moving beyond the asthma algorithm. J Allergy Clin Immunol.

[24] Greene CM, McElvaney NG (2009). Proteases and antiproteases in chronic neutrophilic lung disease—relevance to drug discovery. Br J Pharmacol.

[25] Guilleminault L, Azzopardi N, Arnoult C, Sobilo J, Herve V, Montharu J, Guillon A, Andres C, Herault O, Le Pape A, Diot P, Lemarie E, Paintaud G, Gouilleux-Gruart V, Heuze-Vourc’h N (2014). Fate of inhaled monoclonal antibodies after the deposition of aerosolized particles in the respiratory system. J Control Release.

[26] Haq IJ, Gray MA, Garnett JP, Ward C, and Brodlie M (2015) Airway surface liquid homeostasis in cystic fibrosis: pathophysiology and therapeutic targets. Thorax. 71(3):284-7. 10.1136/thoraxjnl-2015-207588 26719229

[27] Hirsh AJ (2002). Altering airway surface liquid volume: inhalation therapy with amiloride and hyperosmotic agents. Adv Drug Deliv Rev.

[28] Hirsh AJ, Molino BF, Zhang J, Astakhova N, Geiss WB, Sargent BJ, Swenson BD, Usyatinsky A, Wyle MJ, Boucher RC, Smith RT, Zamurs A, Johnson MR (2006). Design, synthesis, and structure-activity relationships of novel 2-substituted pyrazinoylguanidine epithelial sodium channel blockers: drugs for cystic fibrosis and chronic bronchitis. J Med Chem.

[29] Hobbs CA, Blanchard MG, Alijevic O, Tan CD, Kellenberger S, Bencharit S, Cao R, Kesimer M, Walton WG, Henderson AG, Redinbo MR, Stutts MJ, Tarran R (2013). Identification of the SPLUNC1 ENaC-inhibitory domain yields novel strategies to treat sodium hyperabsorption in cystic fibrosis airway epithelial cultures. Am J Physiol Lung Cell Mol Physiol.

[30] Hogg JC, Timens W (2009). The pathology of chronic obstructive pulmonary disease. Annu Rev Pathol.

[31] Holgate ST (2013). Stratified approaches to the treatment of asthma. Br J Clin Pharmacol.

[32] Jakubke HD, Sewald N (2002). Peptides: chemistry and biology.

[33] Kaner Z, Ochayon DE, Shahaf G, Baranovski BM, Bahar N, Mizrahi M, Lewis EC (2015). Acute phase protein alpha1-antitrypsin reduces the bacterial burden in mice by selective modulation of innate cell responses. J Infect Dis.

[34] Kurbatova P, Bessonov N, Volpert V, Tiddens HAWM, Cornu C, Nony P, Caudri D, Grp CW (2015). Model of mucociliary clearance in cystic fibrosis lungs. J Theor Biol.

[35] Labiris NR, Dolovich MB (2003). Pulmonary drug delivery. Part I: physiological factors affecting therapeutic effectiveness of aerosolized medications. Br J Clin Pharmacol.

[36] Lethem MI, James SL, Marriott C, Burke JF (1990). The origin of DNA associated with mucus glycoproteins in cystic fibrosis sputum. Eur Respir J.

[37] Lightwood D, O’Dowd V, Carrington B, Veverka V, Carr MD, Tservistas M, Henry AJ, Smith B, Tyson K, Lamour S, Bracher M, Sarkar K, Turner A, Lawson AD, Bourne T, Gozzard N, Palframan R (2013). The discovery, engineering and characterisation of a highly potent anti-human IL-13 fab fragment designed for administration by inhalation. J Mol Biol.

[38] Livraghi A, Randell SH (2007). Cystic fibrosis and other respiratory diseases of impaired mucus clearance. Toxicol Pathol.

[39] Lopez-Otin C, Matrisian LM (2007). Emerging roles of proteases in tumour suppression. Nat Rev Cancer.

[40] Low TB, Greene CM, O’Neill SJ, McElvaney NG (2011). Quantification and evaluation of the role of antielastin autoantibodies in the emphysematous lung. Pulm Med.

[41] Maehle AH, Prull CR, Halliwell RF (2002). The emergence of the drug receptor theory. Nat Rev Drug Discov.

[42] Maillet A, Guilleminault L, Lemarie E, Lerondel S, Azzopardi N, Montharu J, Congy-Jolivet N, Reverdiau P, Legrain B, Parent C, Douvin DH, Hureaux J, Courty Y, De Monte M, Diot P, Paintaud G, Le Pape A, Watier H, Heuze-Vourc’h N (2011). The airways, a novel route for delivering monoclonal antibodies to treat lung tumors. Pharm Res.

[43] McIvor RA (2015). Emerging therapeutic options for the treatment of patients with symptomatic asthma. Ann Allergy Asthma Immunol.

[44] Melen E, Pershagen G (2012). Pathophysiology of asthma: lessons from genetic research with particular focus on severe asthma. J Intern Med.

[45] Nadel JA (2000). Role of neutrophil elastase in hypersecretion during COPD exacerbations, and proposed therapies. Chest.

[46] Nolan SJ, Thornton J, Murray CS, Dwyer T (2015). Inhaled mannitol for cystic fibrosis. Cochrane Database Syst Rev.

[47] Patton JS, Fishburn CS, Weers JG (2004). The lungs as a portal of entry for systemic drug delivery. Proc Am Thorac Soc.

[48] Perks B, Shute JK (2000). DNA and actin bind and inhibit interleukin-8 function in cystic fibrosis sputa: in vitro effects of mucolytics. Am J Respir Crit Care Med.

[49] Potter J, Matthews LW, Lemm J, Spector S (1960). The composition of pulmonary secretions from patients with and without cystic fibrosis. Am J Dis Child.

[50] Randell SH, Boucher RC (2006). Effective mucus clearance is essential for respiratory health. Am J Respir Cell Mol Biol.

[51] Respaud R, Marchand D, Parent C, Pelat T, Thullier P, Tournamille JF, Viaud-Massuard MC, Diot P, Si-Tahar M, Vecellio L, Heuze-Vourc’h N (2014). Effect of formulation on the stability and aerosol performance of a nebulized antibody. MAbs.

[52] Respaud R, Vecellio L, Diot P, Heuze-Vourc’h N (2015). Nebulization as a delivery method for mAbs in respiratory diseases. Exp Opin Drug Delivery.

[53] Riordan JR (2008). CFTR function and prospects for therapy. Annu Rev Biochem.

[54] Rollins BM, Garcia-Caballero A, Stutts MJ, Tarran R (2010). SPLUNC1 expression reduces surface levels of the epithelial sodium channel (ENaC) in Xenopus laevis oocytes. Channels (Austin).

[55] Rubin BK, Williams RW (2014). Aerosolized antibiotics for non-cystic fibrosis bronchiectasis. Respiration.

[56] Sawicki GS, Chou W, Raimundo K, Trzaskoma B, Konstan MW (2015). Randomized trial of efficacy and safety of dornase alfa delivered by eRapid nebulizer in cystic fibrosis patients. J Cyst Fibros.

[57] Schreiber SL (2000). Target-oriented and diversity-oriented organic synthesis in drug discovery. Science.

[58] Tabrizi MA, Roskos LK (2007). Preclinical and clinical safety of monoclonal antibodies. Drug Discov Today.

[59] Terryah S, Kesimer M, Hill D, Christensen D, Taylor J, and Tarran R (2015) Delivery of short palate lung and nasal epithelial clone 1 (SPLUNC1)-derived peptides to airway surfaces is not impeded by the mucus layer. In: North American Cystic Fibrosis Conference. Phoenix, Arizona

[60] Vasconcellos CA, Allen PG, Wohl ME, Drazen JM, Janmey PA, Stossel TP (1994). Reduction in viscosity of cystic fibrosis sputum in vitro by gelsolin. Science.

[61] Wagener JS, Kupfer O (2012). Dornase alfa (Pulmozyme). Curr Opin Pulm Med.

[62] Xiong MH, Bao Y, Yang XZ, Zhu YH, Wang J (2014). Delivery of antibiotics with polymeric particles. Adv Drug Deliv Rev.

